# Comparison of the surgical outcomes of the posterior approach, video-assisted thoracic surgery, and combined approach for thoracic dumbbell tumors based on a new classification: a retrospective study

**DOI:** 10.1007/s10143-023-02267-y

**Published:** 2024-01-03

**Authors:** Mao Zilong, Zhang Jinan, Li Weixin, Wang Peng, Zuo Wei

**Affiliations:** 1 Department of Spine Surgery, Xi’an No 3. Hospital, the Affiliated Hospital of, Northwest University, Xi’an, 710018 Shannxi China; 2 Department of Neurosurgery, Tangdu Hospital Affiliated Air Force Medical University, Xi’an, 710000 Shannxi China

**Keywords:** Thoracic dumbbell tumors, Classification, Surgical strategy, Video-assisted thoracic surgery

## Abstract

The appropriate surgical treatment strategy was based on the regions of tumor invasion. There is no classification to aid the surgeon in selection. A retrospective study of the clinical data of patients who underwent resection of thoracic dumbbell tumors at the Neurosurgery and Thoracic Surgery Department of Hospital between January 1, 2016, and December 31, 2021 was conducted. Patient data, images, and surgical outcome data were collected. The thoracic spine was divided into areas A, B, and C with respect to the line through the middle of the intervertebral foramen and the line of the costo-transverse joint lateral margin in the horizontal plane. Type I tumors were located in areas A or A and B, type II tumors were located in areas B or B and C, and type III tumors were located in areas A, B, and C. Fifty-five patients with thoracic dumbbell tumors were surgically treated (mean age, 43.1 years; 22 (40%) female). The patients with type I and III tumors underwent the posterior approach, type III tumors had more bleeding during the operation and longer operation times than type I. Among the patients with type II tumors who underwent video-assisted thoracic surgery and the posterior approach, the posterior group had more bleeding and a longer operation time than the others. The patients with type III tumors underwent the combined approach and the posterior approach; although there was no clear difference in the bleeding volume or operation time, the combined approach group had a lower incidence of complications. The new classification of different types of thoracic dumbbell tumors can simply and effectively guide the selection of surgery.

## Introduction

Dumbbell tumors are common, accounting for 13–17% of all spinal tumors, and thoracic dumbbell tumors (TDTs) account for 35% of all dumbbell tumors of the spine [1]. Among thoracic dumbbell tumors, 95% are benign, and approximately 90% are nerve sheath tumors, of which neurofibromas and schwannomas make up the vast majority [2, 3]. TDTs commonly arise from neurogenic elements within the spinal canal or spinal nerve and usually involve the nerve root and encroach on the dura, intervertebral foramen, and paraspinal and thoracic cavities [1, 4, 5]. At present, the primary treatment for dumbbell tumors is surgical removal [6].

A previous study indicated that the posterior approach is a standard surgical approach for excising tumors located in the intraspinal canal and intervertebral foramen [7, 8], thoracic surgery is appropriate for excising tumors located in the thoracic cavity [9], and combined surgery is suitable for removing large tumors [10]. However, the features of TDTs that involve the spinal canal or the thoracic cavity or even both increase the difficulty of selecting the type of TDT that is most suitable for removal by neurosurgeons or thoracic surgeons or which type of TDT can be removed by a combined approach.

However, various classifications of dumbbell tumors have been proposed to guide the selection of surgical strategies for dumbbell tumor resection [7, 11]. The most common classification method included Eden’s, Asazuma’s, and Tong Liu’s systems [7, 12]. Although Eden’s classification was the earliest system proposed and the most widely used, it is limited in guiding the selection of surgical resection. Asazuma’s study focused on cervical dumbbell tumors and neglected lumbar and thoracic spinal dumbbell tumors. Tong Liu proposed a standardized classification to guide the selection of the posterior and combined approaches, but there is no clear description of which types of TDTs are suitable for removal via thoracic surgery or whether one-stage or two-stage combined surgery is suitable.

Consequently, it was necessary to develop a new classification system for TDTs to guide the selection of the best surgical approach. The purposes of this retrospective study are to propose a new classification system and to evaluate the validity of the new classification system to assist surgeons in choosing an appropriate surgical approach.

## Materials and methods

We retrospectively screened all patients who underwent resection of TDTs in the neurosurgery and thoracic surgery departments of the Hospital between January 1, 2016, and December 31, 2021. The patients’ clinical characteristics, imaging results and follow-up outcomes were analyzed. Data, including the surgical procedure duration, blood loss volume, postoperative drainage volume, hospitalization time, and complications (graded according to the Clavien-Dindo classification [13]), were collected. The local ethics board of our institutions approved this study, which was performed in accordance with the ethical standards of the 1964 Declaration of Helsinki. Patient consent was obtained from all patients enrolled in this study. This is an observational study that complies with the STROBE statement.

### Inclusion and exclusion criteria

Patients who underwent surgical resection of tumors invading two or more anatomical regions were considered for inclusion. Patients who had undergone a second surgical resection were excluded. Inclusion criteria: primary tumor; the exclusion criteria: patients with recurrent tumors; patients who were lost to follow up.

### Statistical analysis

SPSS software version 25.0 (SPSS Inc., Chicago, IL) was used for statistical analysis. A difference analysis was performed using the independent *T* test and Mann–Whitney *U* test. Normally distributed data are presented as the means and standard deviations. A *P* < 0.05 was considered statistically significant.

### Surgery

#### Posterior approach

All posterior surgeries were performed by the neurosurgeons in this study. Under general anesthesia, the patient was positioned prone on bolsters. A posterior midline incision was made by neurosurgeons to allow dissection of the subcutaneous soft tissue and muscle and to expose the lamina of centrum, which required resecting the tumor. For extradural tumor invasion, a unitized high-speed drill and rongeur are used to partly excise the lamina, facet, transverse process, and even part of the ribs, until the periphery of the tumor can be observed by microscope. Then, subcapsular resection is performed to remove the tumor, which invaded the intercostal artery and pleura, to protect the artery and pleura from injury. The neurosurgeon carefully divides the capsular of the tumor from the artery and pleura. If it cannot be divided, part of the capsular of the tumor should be preserved. Finally, the neurosurgeon can carefully discern the tumor-bearing nerve and remove it.

In case of intraspinal-extraspinal dumbbell tumors or the extent of the tumor extends beyond the midline, total laminectomy has been performed to expose the spinal canal tumor. The extraspinal tumor also completely exposed by excise facet, transverse process and even part of the ribs thereafter. Under microscope assistance, the intraspinal component should be removed first. A T-shaped incision was made in the dura using 2 steps: the first step was to make a vertical incision along the dura to observe the boundary of tumor, the second was to make a horizontal incision along the nerve root to ensure adequate visibility for complete tumor excision. Then, the neurosurgeon strips the tumor from the spinal cord carefully. The tumor tissue is liberated, leading to gross total tumor excision. Finally, the dura was repaired primarily with running monofilament sutures and the T-shaped was covered with fibrin glue.

#### Video-assisted thoracic surgery

All video-assisted thoracic surgeries (VATSs) were performed by the thoracic surgeon involved in this study. The anesthesiologist performed pulmonary function evaluations in all the patients to identify whether the patients could tolerate collapse of the unilateral lung during the VATS. Patients were administered general anesthesia via a double-lumen endotracheal tube and positioned in a lateral decubitus position. The surgical incision was designed based on the location of the tumor. The ipsilateral lung was collapsed, and three 10-mm portals were made. One camera and two conventional instruments were placed into the thoracic cavity sequentially. The instruments were used to expose and remove the tumor. The tumor was placed in a disposable specimen bag. A chest tube was placed and the lung was reinflated under video guidance. Positive pressure was maintained in the pleura, and the wounds were closed.

#### Combined approach

All combined surgeries were performed by neurosurgeons who first removed the tumors in the spinal canal and then by thoracic surgeons who removed the tumors in the thoracic cavity by thoracotomy.

### Classification

**Fig. 1:**
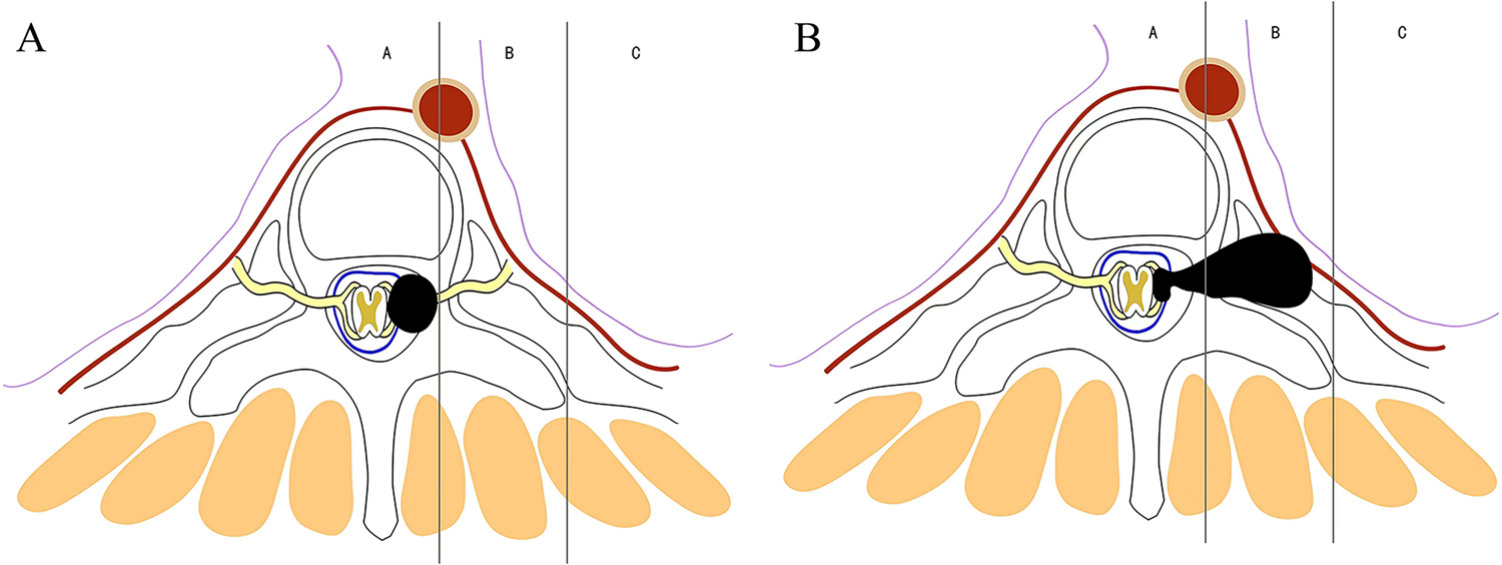
For type I tumors, the largest transverse sections are located in area A or A and B. **A** The tumor was located in area A. **B** The tumor was located in areas A and B

**Fig. 2:**
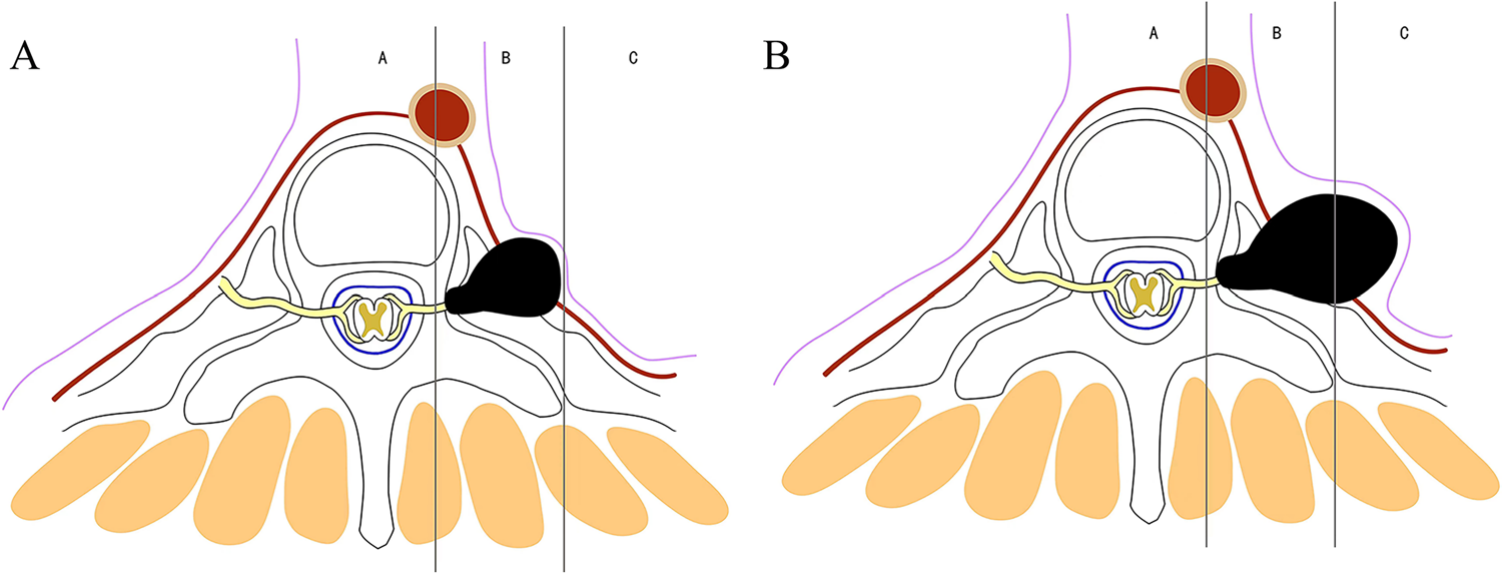
For type II tumors, the largest transverse sections are located in area B or areas B and C. **A** The tumor was located in area B. **B**, The tumor was located in areas B and C

**Fig. 3:**
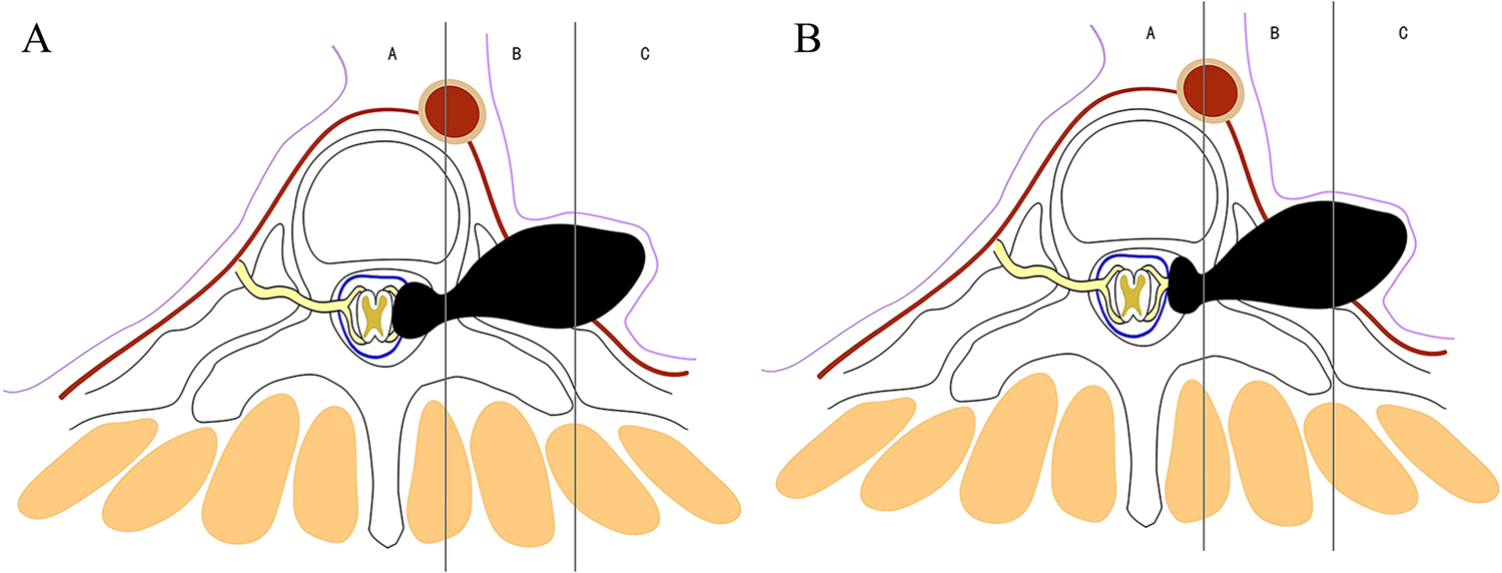
For type III tumors, the largest transverse sections are located in areas A, B, and C. **A** The tumor was intradurally located in the dura and extended to areas B and C. **B** The tumor was not intradurally located in the dura nor extended to areas B and C

Based on the tumor location, degree of tumor spread, tumor characteristics, and anatomical characteristics of the thoracic vertebrae, we propose the classification of thoracic dumbbell tumors. We divided the thoracic vertebrae into three areas (A, B, C) in the horizontal position: area A was medial to the midline in the intervertebral foramen, area B was the area between the midline in the intervertebral foramen, and the vertical line through the costo-transverse joint lateral margin, and area C was lateral to the vertical line through the costo-transverse joint lateral margin. According to these areas, we formulated three types of dumbbell tumors: type I, the largest transverse sections of the tumor were located in area A (Fig. 1A) or both areas A and B (Fig. 1B); type II, the largest transverse sections of the tumor were located in area B (Fig. 2A) or both areas B and C (Fig. 2B); and type III, the largest transverse sections of the tumor were located in areas A, B, and C. In addition, depending on whether the dura was slit to access the intradural component, it was classified into two subtypes: type IIIA (Fig. 3A), in which the intradural dura was removed, and type III B (Fig. 3B), in which there was no need to open the dura.

## Results

Fifty-five patients with TDTs underwent gross total resection of the tumor and the data are shown in Table 1. The baseline demographic data are presented in Table 2. The cohort included 22 female patients (41%), and the mean (SD) age was 43.1 (16.4) years. Of the 55 patients, 2 had a history of hypertension, 2 had a history of diabetes, and 1 had both. Of 55 operations, 29 (53%) were performed using the posterior approach, 23 (42%) were performed using VATS and 3 (5%) were performed using a combined approach (Table 3). The pathology of the tumor is presented in Table 4. Schwannoma was most common (45, 82%).

**Table 1:** The all date of the all patients

Patient no	Age (year)	Sex	Classification	SA	surgery		Complications				Comorbidities	
					BL	OT	PE	PO	PM	CL	HT	D
1	38	M	I	P	300	100						
2	62	F	I	P	20	285						Y
3	32	F	I	P	400	240						
4	40	F	I	P	50	135						
5	38	F	I	P	200	150						
6	49	M	I	P	300	95						
7	62	M	I	P	300	330						
8	59	F	I	P	50	90					Y	
9	61	F	I	P	200	180						
10	27	M	I	P	1600	240						
11	56	M	II	P	300	180					Y	
12	31	M	II	P	100	170						
13	49	M	II	P	300	225						
14	45	M	II	P	150	200						
15	29	M	II	P	100	255						
16	7	M	II	P	30	170						
17	25	M	II	VATS	30	50						
18	32	F	II	VATS	50	60						
19	39	F	II	VATS	50	120						
20	38	M	II	VATS	100	60						
21	43	M	II	VATS	20	60						
22	62	M	II	VATS	10	30						
23	59	M	II	VATS	20	50					Y	Y
24	61	M	II	VATS	20	45						
25	27	M	II	VATS	30	60						
26	56	F	II	VATS	60	80						
27	31	F	II	VATS	20	135						
28	49	F	II	VATS	50	120						
29	45	M	II	VATS	20	100						
30	29	F	II	VATS	30	70						
31	54	F	II	VATS	10	55						
32	49	M	II	VATS	20	55						
33	42	M	II	VATS	10	50						
34	65	F	II	VATS	30	50						
35	28	M	II	VATS	20	120						
36	25	M	II	VATS	100	140						
37	67	M	II	VATS	200	85						
38	28	M	II	VATS	100	75						Y
39	55	F	II	VATS	200	150						
40	54	M	IIIA	P	100	180						
41	41	F	IIIA	P	300	270						
42	33	F	IIIA	CA	1000	420				Y		
43	54	F	IIIA	P	500	240	Y					
44	49	F	IIIA	P	300	270	Y	Y	Y			
45	34	F	IIIA	P	600	380	Y					
46	42	F	IIIA	P	400	300	Y					
47	12	M	IIIB	P	250	205						
48	41	M	IIIB	P	300	210						
49	65	M	IIIB	P	1500	335	Y					
50	48	F	IIIB	P	600	170						
51	28	M	IIIB	P	800	180	Y					
52	25	F	IIIB	P	150	190						
53	67	M	IIIB	CA	250	180						
54	36	M	IIIB	CA	200	190						
55	47	M	IIIB	P	450	385						

*SA* Surgical approach; *P* posterior approach; *VATS* video-assisted thoracic surgery; *CA* combined approach; *BL* blood loss; *OT* operation time; *PE* pleural effusion; *PO*, pneumothorax; *PM* pneumonia; *CL* cerebrospinal fluid leakage; *HT* hypertension; *D* diabetes; *y* yes

**Table 2:** Demographic and summary data for all patients and by study group

	No. (%)					
Characteristics	Type I (*n* = 10)	Type II		Type III		Total
		Neurosurgery (*n* = 6)	Thoracic surgery (*n* = 23)	IIIA (*n* = 7)	III B (*n* = 9)	*n* = 55
Age, mean (SD)	46.8 (13.5)	36.2 (17.7)	44.0 (13.5)	43.9 (8.7)	41.0 (18.1)	43.1 (16.4)
Sex						
Male	4 (40%)	6 (100%)	15 (65%)	1 (14%)	7 (78%)	33 (60%)
Female	6 (60%)	0 (0%)	8 (35%)	6 (86%)	2 (22%)	22 (40%)
Comorbidities						
Hypertension	1 (10%)	1 (17%)	1 (4%)	0	0	3
Diabetes	1 (10%)	0	2 (8%)	0	0	3

**Table 3:** The Classification and surgical approaches (number of cases)

Classification		Posterior approach	VATS	Combined approach
Type I		10	0	0
Type II		6	23	0
Type III	A	6	0	1
	B	7	0	2

**Table 4:** The pathological diagnosis and classification

Pathology	Type I tumor	Type II tumor	Type III tumor
Schwannoma	5	27	13
Neurofibroma	1	2	0
Cysts	3	0	2
Hemangioma	0	0	1
Lipoma	1	0	0

The clinical data of patients with type I and III tumors who underwent the posterior approach and were assessed during surgery are presented in Table 5. Ten type I and thirteen type III tumors were resected via the posterior approach. The patients with type I tumors had less intraoperative bleeding (202.0, 128.9, ml) and shorter operative times (163.5, 63.7, min) than those with type III tumors (480.7, 363.2, ml, 255.0, *P* = 0.03 75.7, min, *P* = 0.06). The patients with type III tumors had a higher incidence of complications; 6 patients (46%; I: 3; II: 2) who underwent the posterior approach experienced pleural effusion, 1 patient 1(7%; II: 1) experienced pneumothorax and 1 patient 1 (7%; II: 1) experienced pneumonia.

**Table 5:** The clinical data of patients with types I and III tumors

Posterior approach	No. (%)		
	Type I tumor (*n* = 10)	Type III tumor (*n* = 13)	*P* value
Surgery characteristics (mean, SD)			
Blood loss (ml)	202.0, 128.9	480.7, 363.2	0.03
Operative time (min)	163.5, 63.7	255.0, 75.7	0.06
Complications no. (%)			
Pleural effusion	0	6(46%; I: 3; II: 3)	
Pneumothorax	0	1(7%; II:1)	
Pneumonia	0	1(7%; II: 1)	

I: Clavien–Dindo classification of grade I; II: Clavien–Dindo classification of grade II

The clinical data of the patients with type II tumors who underwent the posterior approach and VATS were assessed during surgery to assess the surgical outcome are presented in Table 6. Twenty-three VATS procedures were performed and six posterior approach procedures were performed. The patients in the VATS group had less intraoperative bleeding (52.02, 35.1 ml) and shorter operative times (79.1, 35.1 min) than those in the posterior group (163.3, 112.5 ml, *P* = 0.02; 200.0, 34.2 min, *P* = 0.00). The patients in the two groups did not experience surgical complications.

**Table 6:** The clinical data of the patients with type II tumors who underwent the posterior approach and VATS

Type II tumor	No. (%)		
	VATS (*n* = 23)	Posterior approach (*n* = 6)	*P* value
Surgery characteristics (mean, SD)			
Blood loss (ml)	52.2, 35.1	163.3, 112.5	0.02
Operative time (min)	79.1, 35.1	200.0, 34.2	0.00
Hospitalization time (day)	6.9, 1.0	6.8, 0.8	0.86

The clinical data of the patients with type III tumors who underwent the posterior approach and combined approach and were assessed during surgery, and the surgical outcomes are presented in Table 7. Seven cases of type IIIA, including six cases, were treated by the posterior approach, and one case was treated by the combined approach. For the type IIIA patients, there were no distinct differences in the intraoperative bleeding volume (366.7, 175.1, ml; 400.0 ml, *P* = 0.29) and operative time (273.3, 175.1, min; 420.0 ml, *P* = 0.10) between the two groups. The patients in the two groups experienced several surgical complications. The combined approach resulted in several cases of cerebrospinal fluid leakage and pleural effusion. Four patients 4 (67%; I:3; II:1) who underwent the posterior approach experienced several pleural effusions, 1 patient (17%; II:1) experienced pneumothorax, and 1 patient 1 (17%; II:1) experienced a pneumonia.

**Table 7:** The clinical data of the patients with type III tumors who underwent the posterior approach and combined approach

	No. (%)					*P* value
Type III	Type IIIA		*P* value	Type IIIB		
Surgery characteristics (mean, SD)	Posterior approach (*n* = 6)	Combined approach (*n* = 1)		Posterior approach (*n* = 7)	Combined approach (*n* = 2)	
Blood loss (mL)	366.7, 175.1	400.0	0.29	578.6, 462.7	225.0, 35.4	0.33
Operative time (min)	273.3, 63.2	420.0	0.10	239.3, 84.8	185.0, 7.1	0.42
Complication no. (%)						
Pleural effusion	4 (67%; I:3; II:1)	1 (100%; II:1)		1 (14%; II:1)	0	
Pneumothorax	1 (17%; II:1)	0		0	0	
Cerebrospinal fluid leakage	0	1 (100%; II:1)		0	0	
Pneumonia	1 (17%; II:1)	0		0	0	

I: Clavien–Dindo classification of grade I; II: Clavien–Dindo classification of grade II

Nine cases of type IIIB, including seven cases were treated via the posterior approach, and two cases were treated via the combined approach. There were also no distinct differences in intraoperative bleeding volume (578.6, 462.7, ml; 225.0, 35.4 ml, *P* = 0.33) or operative time (239.3, 84.8, min; 185.0, 7.1 ml, *P* = 0.42) between the two groups. The patients who underwent the posterior approach had surgical complications. One patient (14%; II:1) who underwent the posterior approach experienced several pleural effusions.

Among patients undergoing posterior surgery, the patients who underwent unilateral laminectomy and facetectomy received unilateral pedicle screw fixation, and those who underwent bilateral laminectomies and facetectomy underwent bilateral pedicle screw fixation.

Fifty-five patients were followed up in terms of clinical and radiographic outcome variables for 12 to 36 months (mean, 26 months). Fifty-three patients showed clinical improvement, but two patient showed no change in clinical status. There was no evidence of tumor recurrence or internal fixation failure in any of the patients.

## Discussion

### Type I tumor

The type I tumor originated from the spinal canal and did not extend beyond the vertical line through the costo-transverse joint lateral margin. In our 10 cases of type I tumors, all underwent the posterior approach. All patients underwent gross total resection and did not experience complications of surgery. The patients with type III tumors who underwent the posterior approach had more surgical bleeding, longer operative times and higher rates of complications than those with type I tumors.

The advantage of the posterior approach is the removal of tumors located in the spinal canal and intervertebral foramen region. For tumors located at the thoracic paravertebral or intrathoracic region, some surgeons utilize the L-shaped incision, C-shaped incision, or paraspinal approach to obtain vision while removing these tumors [7, 14].Although C- and L-shaped incisions have shorter lengths, amputated thoracic paraspinal muscles cause greater iatrogenic trauma, and the nonstraight incision has poor aesthetics and may hinder wound healing [15]. In addition, some surgeons have utilized the paraspinal approach to remove tumors [16, 17]. The approach has been used more frequently in lumbar spine surgery to reduce the risk of surgical injury. However, in the thoracic spine, the paraspinal muscles are not strong enough and do not have relevance compared with the lumbar spine. Thus, its advantage is nonsignificant compared to the lumbar spine. The approach makes it difficult to excise the tumor located at the spinal canal due to exposure [18, 19].

In our study, we observed that tumors that do not have a costotransverse joint lateral margin can utilize the posterior middle incision to resect tumors. Intraoperatively, the transverse costal joint was utilized as an anatomical marker to expose the tumor margin, and sufficient vision was obtained by rotating the surgical bed and microscope to cause less iatrogenic injury and reduce the risk of intercostal artery and pleural injury.

### Type II tumor

Type II tumors were located outside the midline of the intervertebral foramen. VATS had obvious advantages over the posterior approach in terms of Type II tumors resection. The VATS group had less intraoperative bleeding and a shorter operative time but no significant difference in the length of hospital stays.

Some surgeons have indicated that the most noteworthy advantages of the posterior approach are that no chest drainage system is needed, which can shorten the hospitalization time. However, in our study, the patients who underwent the posterior approach did not show obvious advantages. There are clear advantages for the removal of type II tumors by VATS.

### Type III tumor

Type III tumors arise from the spinal canal and involve the thoracic cavity more than the costo-transverse joint. Whether there was a component of the tumor in the dura mater was classified as type IIIIA or type IIIB. Although the combined approach did not show dominance in bleeding or duration of surgery, the patients who underwent the posterior approach had a higher incidence of pleural effusion.

The combined approach was characterized by changing the patient’s position during the operation and the cooperation of the two teams, leading to increased operation time and intraoperative bleeding. In fact, there was no significant difference between the posterior approach and the combined approach in bleeding volume or time. The combined approach indeed reduces the extent of resection of the facet, transverse process, and the rib and the stripping of the muscle from the spine compared with a single posterior approach.

At present, the general consensus regarding treatment for dumbbell tumors is that components of the intraspinal canal should be removed prior by the neurosurgeon to avoid overstretching the nerve [20–22]. However, whether the combined approach is a one- or two-stage resection has been debated. Compared to the two-stage approach, the one-stage approach is advocated because there is no risk of bleeding from remnant tumor tissue or compression of the spinal cord and hospital costs and the hospitalization time are reduced [21]. The advantages of the two-stage procedure, resection of the intraspinal and paravertebral components on separate occasions, are that both teams are free to operate within their own theatre environment with their own equipment on their own schedule [23]. More importantly, it conforms to the theory of damage control, reduced surgical trauma and accelerated postoperative recovery. For patients who have a weak physical condition and cannot tolerate lengthy surgery, two-stage surgery is likely a better surgical strategy.

Although we lack cases of the two-stage combined approach, the complications of severe pleural effusion and cerebrospinal fluid leakage in one case of a type IIIA tumor indicated that the type IIIA tumor was not suitable for the one-stage combined approach. Resection of type IIIA tumors includes intraspinal and extraspinal communication to the extraforaminal region, requiring that the dura be slit to remove the intraspinal component and the extraforaminal component be removed during thoracic surgery. Damage to the dura and pleura develops into a temporary pipeline, since the negative pressure of the chest leads to continuous cerebrospinal fluid leakage, leading to serious complications. Thus, we propose utilizing the two-stage combined approach to safely and effectively remove type IIIA tumors.

The resection of type IIIB tumors was performed by using a single posterior (*n* = 7) approach and a one-stage (*n* = 2) combined approach. Although the posterior approach had a higher incidence of complications than the combined approach, there was no significant difference in blood loss volume or operative time. Most of the TDTs were benign tumors. The neurosurgeons performed subcapsular resection to facilitate complete tumor removal. Thus, for the resection Type IIIB tumors, we suggest that the neurosurgeons attempt to completely remove the tumor. However, if it cannot be completely removed, the thoracic surgeon should attempt to remove the residual tumor.

### Limitations

Our study has numerous limitations. First, the study was limited by its single-center, retrospective design. Second, the sample size was small because thoracic spinal dumbbell tumors are infrequently encountered. We did not have enough cases to be able to compare the advantages and disadvantages of the posterior approach with those of the combined approach in the resection of type III tumors. In addition, we still need to complete a prospective randomized controlled study to further illustrate the effectiveness and universality of the classification.

## Conclusion

**Fig. 4:**
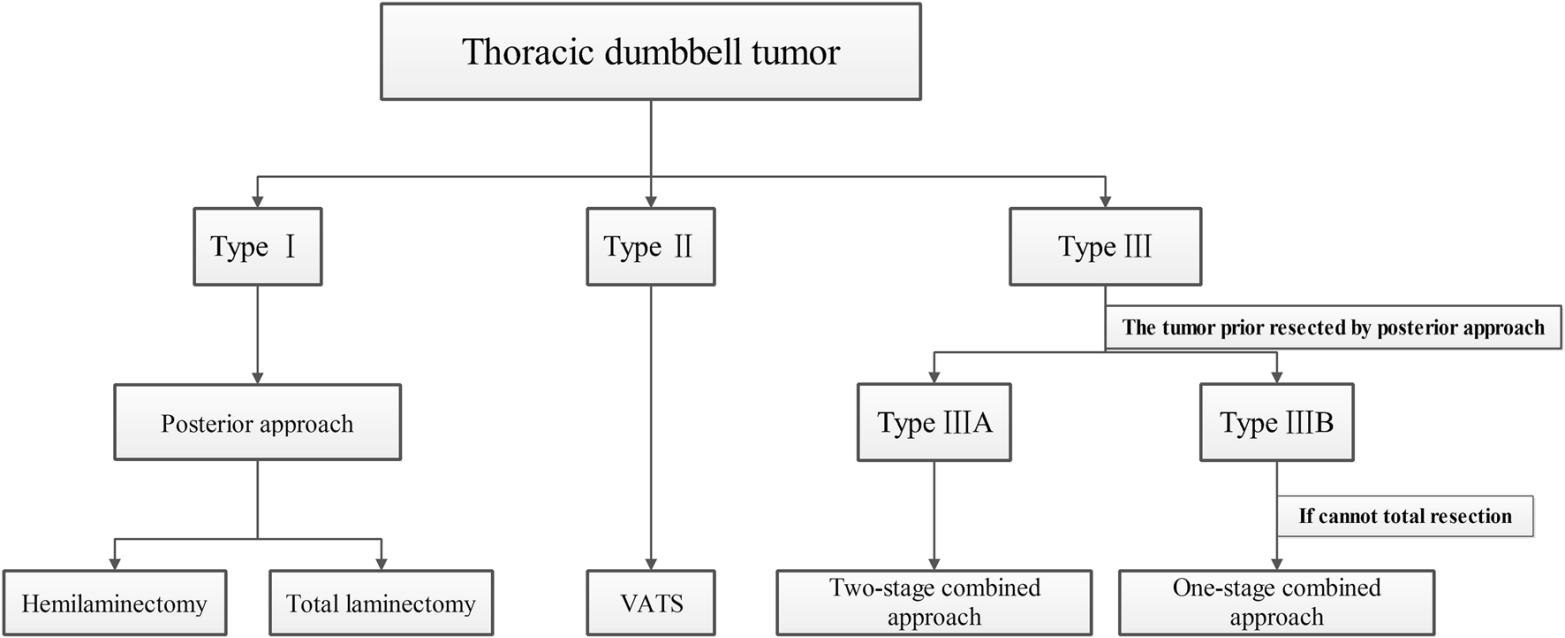
According to the classification, an appropriate surgical strategy was selected

According to the classification we proposed, selecting an appropriate surgical strategy was indispensable for achieving safe, complete resection of the tumor while causing minimal trauma. The posterior approach was suitable for removing type I tumors. There is no doubt that a type II tumor can be excised using VATS. The two-stage approach is better for the removal of type IIIA tumors. For type IIIB tumors, we suggest that the neurosurgeons attempt to completely remove the tumor. If they cannot, the thoracic surgeon should attempt to resect the residual tumor (Fig. 4).

## Data Availability

The all date was included in the manuscripts.
